# The BET bromodomain inhibitor ZEN-3365 targets the Hedgehog signaling pathway in acute myeloid leukemia

**DOI:** 10.1007/s00277-021-04602-z

**Published:** 2021-08-01

**Authors:** Jasmin Wellbrock, Lena Behrmann, Jana Muschhammer, Franziska Modemann, Kais Khoury, Franziska Brauneck, Carsten Bokemeyer, Eric Campeau, Walter Fiedler

**Affiliations:** 1 Department of Oncology, Hematology and Bone Marrow Transplantation With Section Pneumology, Hubertus Wald University Cancer Center, University Medical Center Hamburg Eppendorf, Martinistrasse 52, 20246 Hamburg, Germany; 2 Zenith Epigenetics Ltd, 4820 Richard Road SW, Suite 300, Calgary, AB T3E 6L1 Canada

**Keywords:** Acute myeloid leukemia, Hedgehog, BET inhibitor, GANT-61, ZEN-3365, GLI

## Abstract

Modern cancer therapies increased the survival rates of acute myeloid leukemia (AML) patients tremendously. However, the complexity of the disease and the identification of new targets require the adaptation of treatment protocols to reduce side effects and increase benefit for the patients. One key regulator of leukemogenesis and chemotherapy resistance in AML is the Hedgehog (HH) signaling pathway. It is deregulated in numerous cancer entities and inhibition of its downstream transcription factors GLI translates into anti-leukemic effects. One major regulator of GLI is BRD4, a BET family member with epigenetic functions. We investigated the effect of ZEN-3365, a novel BRD4 inhibitor, on AML cells in regard to the HH pathway. We show that ZEN-3365 alone or in combination with GANT-61 reduced GLI promoter activity, cell proliferation and colony formation in AML cell lines and primary cells. Our findings strongly support the evaluation of the BRD4 inhibitor ZEN-3365 as a new therapeutic option in AML.

## Introduction

Modern chemotherapeutics and newly developed personalized and targeted therapies enhanced the overall survival of cancer patients tremendously within the last decades. However, acute myeloid leukemia (AML) remains as a highly complex and heterogeneous disorder with still insufficient survival rates. This is especially true for elderly patients [1]. Thus, new therapeutic options are necessary to improve the overall outcome and to reduce the fatal side effects of chemotoxic treatments.

Due to the frequently occurring dysregulations of the Hedgehog (HH) signaling cascade in cancer, it has become an interesting target for therapeutic interventions. HH signaling plays a key role in embryogenesis and stem cell maintenance. It is activated by ligand binding to the cell surface receptor Patched (PTCH) that normally inhibits Smoothened (SMO), which mediates downstream signal transduction towards the GLI trancription factors (GLI1-3) [2]. Several HH inhibitors were tested in clinical trials for the treatment of different cancer entities, mainly with the focus on SMO inhibition. SMO inhibitors such as vismodegib, IPI-926, and sonidegib showed high efficacy in cancer types like basal cell carcinoma or medulloblastoma which often harbor activating mutations in the HH pathway [3]. For hematologic malignancies, glasdegib showed in combination with low-dose cytarabine significant higher complete response rate and longer overall survival than cytarabine alone, leading to an FDA approval in 2020 [4].

However, the Hedgehog cascade can also be activated non-canonically through a variety of pathways including the TGF-β, PI3K/AKT/mTOR, and RAS/RAF/MEK/MAPK cascades [5]. Most of those pathways lead to a direct activation of the HH transcription factors GLI1 or GLI2 and thus they signal independently of HH ligands or receptors. We could recently show that expression of HH pathway transcription factors GLI1 and GLI2 represents a negative prognostic marker for acute myeloid leukemia and that targeted inhibition of GLI1/2 mediates anti-leukemic effects in vitro and in vivo [6]. Besides, we revealed that the HH pathway activation in FLT3-mutated AML is partly mediated in a non-canonical way [7]. Therefore, in comparison to SMO, GLI transcription factors might represent more promising therapeutic targets as their inhibition will interrupt the canonical as well as non-canonical signaling. But so far, specific GLI inhibitors like GANT-61 have not entered the clinical stage [8].

In the recent years, targeting epigenetic regulators had come into focus as therapeutic strategy in oncology. Bromodomain and extraterminal domain (BET) family members represent one group of epigenetic modulators regulating gene transcription through recruitment of regulatory complexes to acetylated chromatin regions and interaction with RNA polymerase II [9]. The BET family member BRD4 was shown to regulate the transcription of important proto-oncogenes including MYC or BCL2 and mediates the repression of the tumor suppressor p53 [10, 11]. BRD4 was identified as promising target structure in acute myeloid leukemia as inhibition of BRD4 using shRNA or a small molecule inhibitor resulted in anti-leukemic effects in vitro as well as in vivo [12]. Recently, a direct link between BRD4 and the Hedgehog pathway was identified as two independent studies revealed that BRD4 can directly bind to the promoters of GLI1 and GLI2 thereby regulating their transcription [13, 14].

Here we examined the impact of BRD4 inhibition on GLI signaling in AML. Utilizing the new BRD4 inhibitor ZEN-3365, we could show a significant role of epigenetic regulation for AML cell survival. Furthermore, we have found that GLI inhibition via GANT-61 and the combinatorial blocking of BRD4 have a strong effect on AML cell proliferation and colony forming capacities. Thus, our data provide the evidence that the BET inhibitor ZEN-3365 should be evaluated as therapeutic option in AML.

## Materials and methods

### Cell lines and cell culture

The following human AML cell lines were used: MV4-11 (ATCC, #CRL9591, RRID:CVCL_0064), MOLM13 (DSMZ, #ACC554, RRID:CVCL_2119), HL60 (DSMZ, #ACC3, RRID:CVCL_002), KG-1 (DSMZ, #ACC14, RRID:CVCL_0374), Kasumi-1 (DSMZ, #ACC220, RRID_CVCL_0589), OCI-AML3 (DSMZ, #ACC582, RRID:CVCL_1844). Verifications were performed with multiplex human cell line authentification test (MCA) from multiplexion. MV4-11, MOLM-13, HL60, and KG-1 were cultured in RPMI-1640 medium (Gibco) supplemented with 10% fetal bovine serum (FBS, Biochrom GmbH). The AML cell line Kasumi-1 was cultured in RPMI-1640 medium supplemented with 20% FBS. The AML cell line OCI-AML3 was cultured in α-MEM medium (Gibco) supplemented with 20% FBS. Primary AML cells were obtained after patient’s informed consent and approval of the study by the ethics committee (PV3469, Ethik-Kommission der Ärztekammer Hamburg). Cells were isolated from bone marrow using density gradient centrifugation and cultured as described elsewhere [15]. All cells were maintained in a humidified incubator with 5% CO_2_ at 37 °C.

### Protein isolation and western blot analysis

Proteins of MV4-11, MOLM-13, and OCI-AML3 cells were extracted using the trichloroacetic acid method. Protein extracts were applied to a 4–20% SDS-PAGE (Thermo Fisher Scientific, Rockford, IL) followed by electrotransfer to nitrocellulose membranes (Schleicher & Schuell, Dassel, Germany). Blots were incubated with either rabbit anti-human GLI1 (C68H3, Cell Signaling Technology, RRID:AB_1903989) or mouse anti-human β-Actin (sc-47778, Santa Cruz Biotechnology, RRID:AB_2714189) at 4 °C overnight. The subsequent incubation with the peroxidase-conjugated secondary antibodies (anti-mouse IgG, NXA931, GE healthcare, RRID:AB_772209 and anti-rabbit IgG, 7074S, Cell Signaling Technology, RRID:AB_2099233) was followed by detection using ECL Western blotting detection reagents (GE Healthcare) and the FusionSL 4 3500 WL detection system (Vilber Lourmat, Sud Torcy, France).

### GLI reporter assays

Stable GLI reporter AML cell lines were produced using lentiviral particles containing the firefly luciferase gene under the control of GLI1 and GLI 2 transcriptional response elements and as internal control the renilla luciferase gene under CMV promoter elements (CignalTM Lenti Reporters, Qiagen) followed by puromycin and hygromycin selection. Stable GLI reporter cells were treated with ZEN-3365 and the GLI promoter activity was measured after 24 h or at different time-points up to 84 h using the Dual-GLO Luciferase Assay Kit (Promega) and the Infinite F200 PRO reader (Tecan). The firefly luciferase-mediated GLI promoter activity was normalized to the renilla luciferase-mediated CMV promoter activity.

### Proliferation assay

AML cell lines or primary AML blasts were cultured with different concentrations of the GLI inhibitor GANT-61 (2,20-[[Dihydro-2-(4-pyridinyl)-1,3(2H,4H)-pyrimidinediyl]bis(methylene)]bis[N,N-dimethylbenzenamine]; Tocris Bioscience) or the BET inhibitor ZEN-3365 (Zenith Epigenetics) either alone or in combination or dimethyl sulfoxide (DMSO, Sigma-Aldrich) as solvent control. Cell numbers were determined after 3 and 7 days using the cell viability analyzer Vi-Cell TM XR (Beckman Coulter).

### Colony formation assay

AML cell lines were seeded with GANT-61, ZEN-3365, or their combination in Methocult (Methocult H4230, Stemcell Technologies). DMSO was used as solvent control. The number of colonies was counted after 7 days using an inverted microscope (Axiovert 25, Zeiss) and normalized to DMSO control.

### Statistical analysis

Figures were created with GraphPad PRISM version 6.2. Data was statistically analy**z**ed by Welch**’**s t-test (SPSS Inc.). A *P* value less than 0.05 was considered to be statistically significant.

## Results

### Reduced GLI levels upon treatment with the BET inhibitor ZEN-3365

**Fig. 1:**
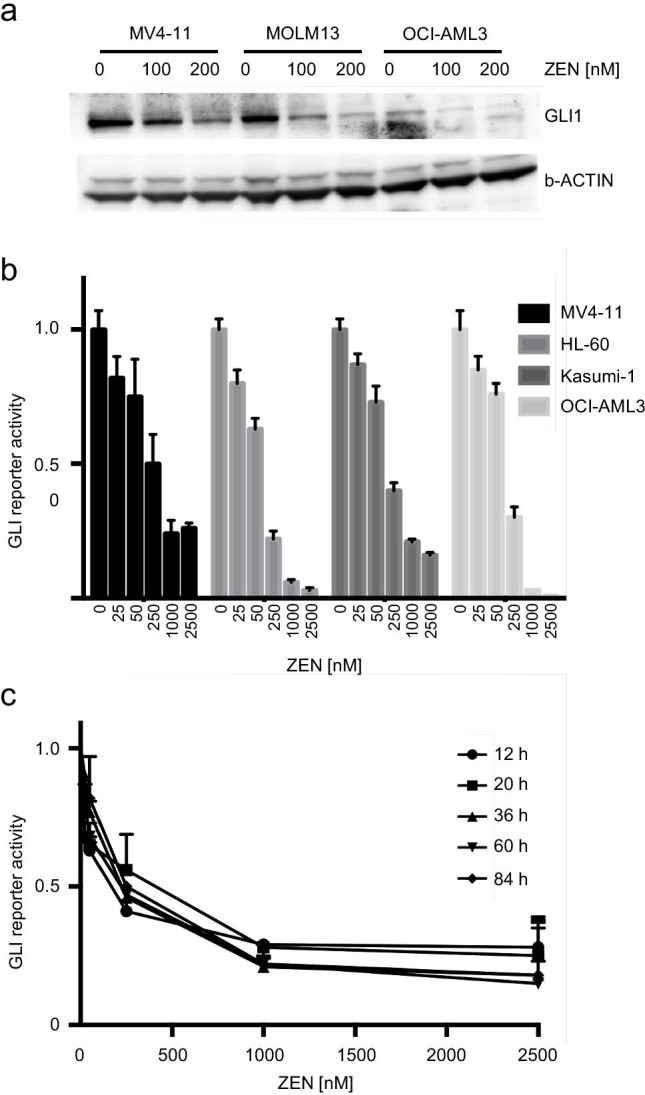
BET inhibitor ZEN-3365 reduces GLI reporter activity in vitro. **a** Western blot of GLI protein levels under ZEN-3365 treatment. MV4-11, MOLM13, and OCI-AML3 were cultivated for 3 days with 100 or 200 nM ZEN-3365. **b** GLI promoter assays reveal the dependence of GLI expression and BRD4 regulation. MV4-11, HL-60, Kasumi-1, and OCI-AML3 were treated with different concentrations of ZEN-3365 (25 to 2500 nM) for 24 h. **c** Time course experiment for BRD4 inhibition with 25, 50, 250, 1000, and 2500 nM ZEN-3365 on MV4-11 cells reveals a stable inhibitory function over 4 days

First, we investigated the impact of the BET bromodomain inhibitor ZEN-3365 on the expression on the HH transcription factors GLI using immunoblotting and GLI reporter assays. For western blot analysis, AML cell lines MV4-11, MOLM13, and OCI-AML3 were incubated with 100 or 200 nM ZEN-3365 for 3 days. ZEN-3365 treatment led to a dose-dependent decrease of the GLI1 protein expression in all three cell lines (Fig. 1a). Next, we generated GLI reporter cell lines carrying the luciferase gene under the control of the GLI promoter which allows the determination of the GLI promoter activity by measuring the bioluminescence signal after addition of a luciferase substrate. The reporter cells were treated with increasing concentrations of ZEN-3365. The GLI reporter activity was measured after 24 h. As shown in Fig. 1b, ZEN-3365 led to a markedly and dose-dependent decrease of the GLI reporter activity in all analyzed cell lines. A time course experiment over 4 days revealed that the dose-dependent inhibitory effects of ZEN-3365 on GLI are stable over time as shown for MV4-11 cells in Fig. 1c.

### The combination of ZEN-3365 with GANT-61 further reduces the GLI promoter activity

**Fig. 2:**
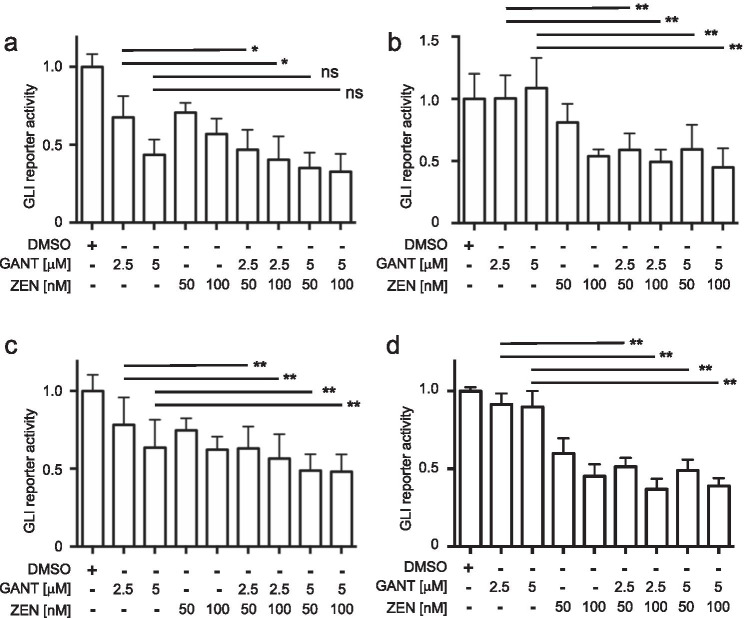
Combinatorial treatment of AML reporter cell lines with ZEN-3365 and GANT-61 decreases GLI reporter activity in vitro. **a** MV4-11, **b** MOLM13, **c** Kasumi-1, and **d** HL60 reporter cell lines were treated with 2500 or 5000 nM GANT-61 and 50 nM or 100 nM ZEN-3365 for 24 h. Significance calculated against GANT-61 single treatment, *p < 0.05, **p < 0.01, and ns = not significant

So far, direct GLI antagonists such as GANT-61 have not entered clinical stages yet. Nevertheless, combination therapies might represent a promising clinical strategy. Therefore, we investigated whether the combination of GANT-61 with ZEN-3365 augmented the inhibitory effect on the HH signaling using the GLI reporter cell lines. The AML cell lines MV4-11, MOLM13, Kasumi-1, and HL60 were treated with 2500 or 5000 nM of GANT-61 and 50 or 100 nM of ZEN-3365, the combinations of both substances or the solvent control. The GLI reporter activity was measured after 24 h. The combination of GANT-61 with ZEN-3365 resulted in a significantly decreased GLI reporter activity in all analyzed cell lines. While this effect was only observed for the low GANT-61 concentration in MV4-11 (Fig. 2a), the combinations of all concentrations led to a significantly reduced GLI reporter activity in MOLM13, Kasumi-1, and HL60 cells compared to GANT-61 treatment alone (Fig. 2b–d).

### The combination of ZEN-3365 and GANT-61 results in decreased leukemic proliferation

**Fig. 3:**
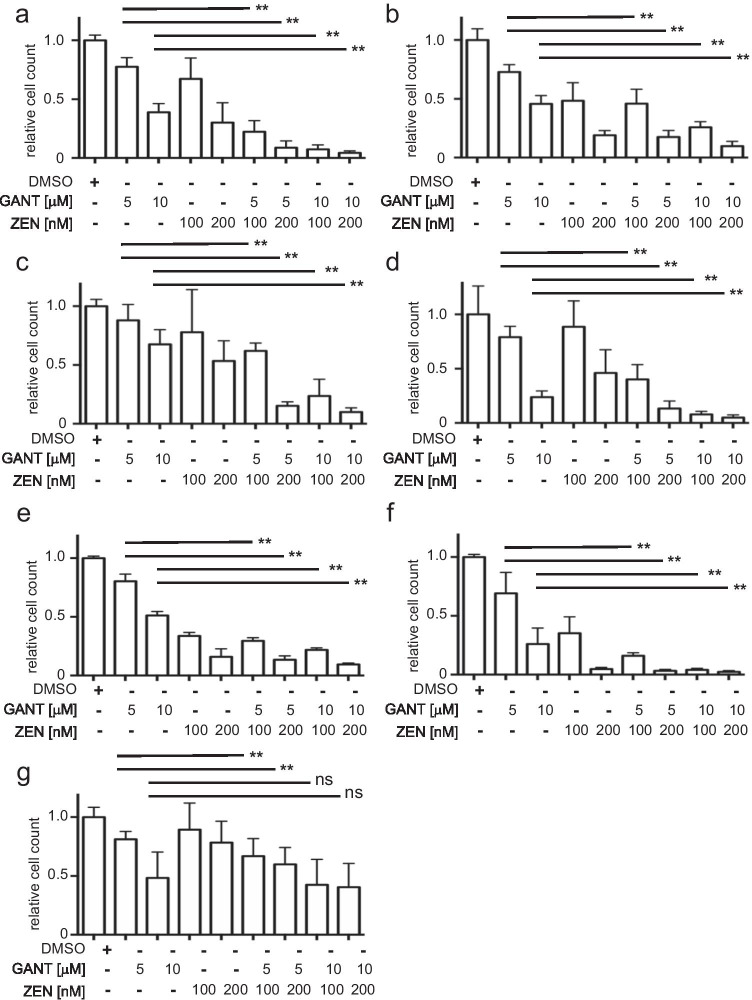
ZEN-3365 and GANT-61 present antiproliferative effects. GANT-61 (5 or 10 μM) and ZEN-3365 (100 or 200 nM) inhibit cell proliferation over 7 days for **a** MV4-11, **b** HL60, **c** KG-1, **d** MOLM13, **e** Kasumi-1, and **f** OCI-AML3. **g** Combinatorial treatment of primary AML sample with GANT-61 (5 or 10 M) and ZEN-3365 (100 or 200 nM) leads to a decreased cell proliferation after 4 days. Significance calculated against GANT-61 treatment alone, *p<0.05, **p<0.01, and ns=not significant

Next, the influence of the ZEN-3365 and GANT-61 combination on the growth and survival of AML cells was investigated in proliferation assays. The combination of 5 or 10 µM GANT-61 with 100 or 200 nM ZEN-3365 led to a significantly reduced growth of the AML cell lines MV4-11, HL60, KG-1, MOLM13, Kasumi-1, and OCI-AML3 in comparison to GANT-61 treatment alone (Fig. 3a–f). Furthermore, primary AML blasts from nine newly diagnosed AML patients were also analyzed for their response to the ZEN-3365 and GANT-61 combination. Figure 3g shows the average proliferation rate of all tested primary AML samples on day 4 under treatment of 100 and 200 nM ZEN-3365 in combination with 5 and 10 µM GANT-61, resulting also in a significant decrease of viable cells.

### Inhibition of GLI signaling via ZEN- 3365 and GANT-61 decreases the progenitor capacities of AML cell lines

**Fig. 4:**
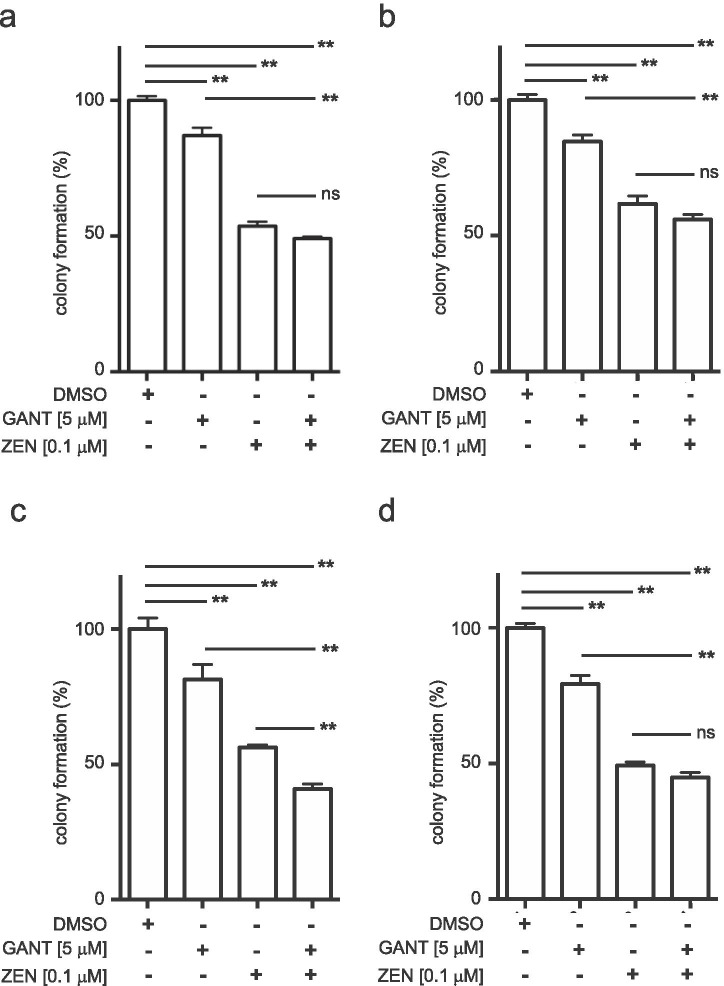
The colony formation potential of AML cell lines is decreased under single or combinatorial treatment. **a** MV4-11, **b** MOLM13, **c** OCI-AML3, and **d** HL60 were treated with 5 μM GANT-61 and 0.1 μM ZEN-3365 or in combination and cultured in methocult medium for one week. Colony numbers were counted with an inverted microscope. *p<0.05, **p<0.01, and ns=not significant

Subfractions of AML cell lines have the potential to develop colonies in semisolid methylcellulose medium, comparable to hematopoietic stem and progenitor cells. The number of colonies is proportional to the size of the subfraction with progenitor capacities. Since HH signaling is regulating the development, maintenance, and expansion of leukemic stem cells [16], we analyzed the effect of the combined GANT-61 and ZEN-3365 treatment with MV4-11, MOLM13, OCI-AML3, and HL60 cells in colony formation assays. As shown in Fig. 4a–d, single treatments with 5 µM GANT-61 and 0.1 µM ZEN-3365 led to a significantly reduced colony number compared to solvent controls (Fig. 4a–d). The combination of both drugs showed significant less colonies in comparison to ZEN-3365 only for OCI-AML3 (Fig. 4c).

## Discussion

In the last decade, numerous new treatment strategies for AML were developed, representing a significant advantage in the therapeutic options for leukemic patients. However, emergence of resistant clones enables the clinical recurrence of the disease, leading frequently to fatal outcome for the individual patient [1]. A key driver of disease development is the leukemic stem cell (LSC) population in AML that plays a critical role in pathogenesis, progression, and resistance against chemotherapy. The identification of LSCs and their role in AML pathogenesis and treatment failure put them into focus of research to identify new agents that can eradicate the LSC population and consequently the AML disease [17]. An interesting target for LSC eradication is the components of the HH signaling pathway, a highly conserved pathway that is known to play a key role in LSC maintenance and expansion [18]. GLI represents a downstream transcription factor family, activated by both, the canonical and the non-canonical HH signaling cascade [19]. GLI overexpression was reported for LSCs and treatment with GANT-61 induced apoptosis in combination with cytarabine and rapamycin [20, 21]. However, clinical application of GANT-61 is difficult since micromolar concentrations are required, leading to higher toxicity and halflife time is short because of rapid hydrolyzation. New direct GLI antagonists like glabrescione B or 8-hydroxyquinoline from Dash et al. represent more promising compounds for clinical testing [22].

Here, we show for the first time that the BRD4 inhibitor ZEN-3365 can reduce the activity of the Hedgehog signaling cascade through inhibition of the GLI transcription factors in AML cells. Furthermore, the combined treatment of AML cells with ZEN-3365 and GANT-61 resulted in significantly reduced cell growth and colony formation capacity compared to the single-agent treatment, leading to the conclusion that BRD4 inhibition with ZEN-3365 represents a promising strategy for rational combinatorial AML therapy.

BRD4, a part of the BET protein family, is known as an epigenetic reader for acetylated histones and regulates global transcription [10]. In AML, it was identified as a modifier of c-Myc expression in MLL-rearranged leukemia in a functional genomics screening, representing a critical factor for disease maintenance [23]. The anti-leukemic effects of BET protein inhibition could also be confirmed in non-MLL-arranged AML subtypes in several in vitro studies [24, 25]. Interestingly, JQ1, an pharmacological inhibitor of BRD4, is able to delay disease progression in AML-bearing mice at doses that showed only minimal effects on normal hematopoiesis [23]. Furthermore, JQ1 has the potential to decrease the number of leukemic stem and progenitor cells which is important to reduce the risk of relapse [23]. The treatment of multiple myeloma cells with the BRD4 inhibitor JQ1 resulted in the loss of BRD4 at the majority (90%) of promoters and more than half (60%) of the enhancer regions with a preferential loss of BRD4 at super-enhancers of key oncogenic drivers such as c-MYC [26]. Interestingly in glioblastoma multiforme, GLI2 was among the super-enhancer-associated genes [26]. Furthermore, several hematopoietic transcription factors (ERG, FLI1, PU.1, C/EBPa, C/EBPb, MYB) use the lysine acetyltransferase p300 as coactivator for the recruitment of BRD4. Interestingly, p300 is also involved in the regulation of the GLI transcription factors as it can acetylate GLI1 and GLI2 thereby decreasing the GLI activity [27, 28].

Previous studies have shown that BET inhibitors such as OTX015 attenuate the mRNA and protein expression of several oncogenes including c-MYC, BCL-2, or CDK6 on the one hand and induce cell differentiation via induction of p21 or HEXIM1 [29–31]. At present, the BRD4 inhibitors PLX51107, OTX015, and GSK525762 are evaluated in clinical trials for refractory/relapsed AML. But despite promising preclinical tests, their efficacy for AML treatment as single agents is modest [12].

Therefore, several combinations of BET inhibitors with other targeted therapies have been investigated. Saenz et al. described synergistic effects when combining BET inhibitors with the Jak2 inhibitor ruxolitinib in post-MPN sAML cells [30, 31]. Furthermore, these post-MPN sAML cells show a higher sensitivity to PROTAC (proteolysis-targeting chimera) ARV-825 compared to normal hematopoietic stem and progenitor cells, enabling an important window of therapy [31]. Most recently, Latif et al. reported enhanced toxicity of BETi and MDM2i in AML cells and in vivo mouse models [11]. The combination of BRD4 and MDM2 inhibition leads to an increase of p53 activation and its pro-apoptotic functions.

Increased anti-leukemic effects have also been observed upon combination of BET inhibitors with FLT3 kinase inhibitors in FLT3-mutated AML cells [32]. In our previously published work, we could identify a non-canonical link between FLT3 signaling and the activation of the Hedgehog transcription factors GLI1 and GLI2 [6, 7]. The occurrence of FLT3 mutations was correlated with high expression of GLI1 and GLI2 and the combined treatment of FLT3 signaling inhibitors and GANT-61 resulted in increased anti-leukemic effects. Hence, the high response rates of FLT3-mutated AML cells to BET inhibitors might in part be due to the FLT3-mediated GLI activation in this AML subgroup.

In summary, AML is a heterogenous disease with aberant regulations of numerous pathways referable to genetic or epigenetic abnormalities. Among them, the HH signaling pathway represents a promising target for cancer therapy, especially the downstream transcription factors of the GLI family. Our results emphasize that the specific inhibition of BRD4 by ZEN-3365 leads to a decrease of GLI expression and a downregulation of HH signaling, resulting in a decrease of proliferation and colony formation potential. Future in vivo studies have to confirm our findings, proving the promising anti-leukemic potential of ZEN-3365 as a single drug but also in combination with other chemotherapeutic applications.

## Data Availability

Not applicable.

## References

[1] Döhner H, Weisdorf DJ, Bloomfield CD (2015). Acute myeloid leukemia. N Engl J Med.

[2] McMillan R, Matsui W (2012). Molecular pathways: the hedgehog signaling pathway in cancer. Clin Cancer Res.

[3] Xie H, Paradise BD, Ma WW, Fernandez-Zapico ME (2019) Recent advances in the clinical targeting of Hedgehog/GLI signaling in cancer. Cells 8:394:1–17. 10.3390/cells8050394 10.3390/cells8050394 PMC6562674 31035664

[4] Cortes JE, Heidel FH, Hellmann A (2019). Randomized comparison of low dose cytarabine with or without glasdegib in patients with newly diagnosed acute myeloid leukemia or high-risk myelodysplastic syndrome. Leukemia.

[5] Pietrobono S, Gagliardi S, Stecca B (2019) Non-canonical Hedgehog signaling pathway in cancer: activation of GLI transcription factors beyond smoothened. Front Genet 10:556:1–20. 10.3389/fgene.2019.00556 10.3389/fgene.2019.00556 PMC6581679 31244888

[6] Wellbrock J, Latuske E, Köhler J (2015). Expression of Hedgehog pathway mediator GLI represents a negative prognostic marker in human acute myeloid leukemia and its inhibition exerts antileukemic effects. Clin Cancer Res.

[7] Latuske E-M, Stamm H, Klokow M (2017). Combined inhibition of GLI and FLT3 signaling leads to effective anti-leukemic effects in human acute myeloid leukemia. Oncotarget.

[8] Terao T, Minami Y (2019) Targeting Hedgehog (Hh) pathway for the acute myeloid leukemia treatment. Cells 8:312:1–11. 10.3390/cells8040312 10.3390/cells8040312 PMC6523210 30987263

[9] Shi J, Vakoc CR (2014). The mechanisms behind the therapeutic activity of BET bromodomain inhibition. Mol Cell.

[10] Spriano F, Stathis A, Bertoni F (2020). Targeting BET bromodomain proteins in cancer: the example of lymphomas. Pharmacol Ther.

[11] Latif A-L, Newcombe A, Li S (2021). BRD4-mediated repression of p53 is a target for combination therapy in AML. Nat Commun.

[12] Kirtonia A, Pandya G, Sethi G (2020). A comprehensive review of genetic alterations and molecular targeted therapies for the implementation of personalized medicine in acute myeloid leukemia. J Mol Med.

[13] Tang Y, Gholamin S, Schubert S (2014). Epigenetic targeting of Hedgehog pathway transcriptional output through BET bromodomain inhibition. Nat Med.

[14] Long J, Li B, Rodriguez-Blanco J (2014). The BET bromodomain inhibitor I-BET151 acts downstream of smoothened protein to abrogate the growth of hedgehog protein-driven cancers. J Biol Chem.

[15] Pabst C, Krosl J, Fares I (2014). Identification of small molecules that support human leukemia stem cell activity ex vivo. Nat Methods.

[16] Ok CY, Singh RR, Vega F (2012). Aberrant activation of the hedgehog signaling pathway in malignant hematological neoplasms. Am J Pathol.

[17] Pollyea DA, Jordan CT (2017). Therapeutic targeting of acute myeloid leukemia stem cells. Blood.

[18] Campbell V, Copland M (2015). Hedgehog signaling in cancer stem cells: a focus on hematological cancers. Stem Cells Cloning.

[19] Jamieson C, Martinelli G, Papayannidis C, Cortes JE (2020). Hedgehog pathway inhibitors: a new therapeutic class for the treatment of acute myeloid leukemia. Blood Cancer Discov.

[20] Long B, Wang L-X, Zheng F-M (2016). Targeting GLI1 suppresses cell growth and enhances chemosensitivity in CD34+ enriched acute myeloid leukemia progenitor cells. Cell Physiol Biochem.

[21] Pan D, Li Y, Li Z (2012). Gli inhibitor GANT61 causes apoptosis in myeloid leukemia cells and acts in synergy with rapamycin. Leuk Res.

[22] Dash RC, Wen J, Zaino AM (2021). Structure-based virtual screening identifies an 8-hydroxyquinoline as a small molecule GLI1 inhibitor. Mol Ther Oncolytics.

[23] Zuber J, Shi J, Wang E (2011). RNAi screen identifies Brd4 as a therapeutic target in acute myeloid leukaemia. Nature.

[24] Dawson MA, Gudgin EJ, Horton SJ (2014). Recurrent mutations, including NPM1c, activate a BRD4-dependent core transcriptional program in acute myeloid leukemia. Leukemia.

[25] Chen C, Liu Y, Lu C (2013). Cancer-associated IDH2 mutants drive an acute myeloid leukemia that is susceptible to Brd4 inhibition. Genes Dev.

[26] Lovén J, Hoke HA, Lin CY (2013). Selective inhibition of tumor oncogenes by disruption of super-enhancers. Cell.

[27] Canettieri G, Di Marcotullio L, Greco A (2010). Histone deacetylase and Cullin3-REN(KCTD11) ubiquitin ligase interplay regulates Hedgehog signalling through Gli acetylation. Nat Cell Biol.

[28] Coni S, Antonucci L, D’Amico D (2013). Gli2 acetylation at lysine 757 regulates hedgehog-dependent transcriptional output by preventing its promoter occupancy. PLoS ONE.

[29] Khan M, Siddiqi R, Gangat N (2017). Therapeutic options for leukemic transformation in patients with myeloproliferative neoplasms. Leuk Res.

[30] Saenz DT, Fiskus W, Manshouri T (2017). BET protein bromodomain inhibitor-based combinations are highly active against post-myeloproliferative neoplasm secondary AML cells. Leukemia.

[31] Saenz DT, Fiskus W, Qian Y (2017). Novel BET protein proteolysis-targeting chimera exerts superior lethal activity than bromodomain inhibitor (BETi) against post-myeloproliferative neoplasm secondary (s) AML cells. Leukemia.

[32] Fiskus W, Sharma S, Qi J (2014). BET protein antagonist JQ1 is synergistically lethal with FLT3 tyrosine kinase inhibitor (TKI) and overcomes resistance to FLT3-TKI in AML cells expressing FLT-ITD. Mol Cancer Ther.

